# Supplementary material for: MicroRNA-379 Modulates Prostate-Specific Antigen Expression Through Targeting the Androgen Receptor in Prostate Cancer
Source: Cancers (Basel). 2025 Oct 7;17(19):3245. doi: 10.3390/cancers17193245 (PMC12524244; doi:10.3390/cancers17193245)
Supplement: Supplementary file 1 [file cancers-17-03245-s001.zip › Supplementary/Supplementary legends.pdf]

**Supplementary Figure S1.** Repeated Cytokine array**.** 22Rv1 cells with downregulated miR-379 compared to a Scr control grown in OBCM. The raw blots **(a)** are shown with spots corresponding to PSA highlighted in orange. The waterfall plot **(b)** shows the positive fold change increase in cytokines from 22Rv1 cells grown in OBCM. PSA is highlighted in the orange bar.

**Supplementary Figure S2.** DELFIA Secreted PSA. Both total and free PSA was measured in supernatants from VCaP cells. The left bar negative control-treated cells and the right bar denotes miR-379 cells in normal media. Unpaired two-tailed Student's *t*-tests were performed to compare the treatment groups to one another; $*p < 0.05$. Only statistically significant *p* values are shown in the figure. Experiments were performed three times and representative data is shown.

**Supplementary Figure S3.** Luciferase reporter assays**.** The left bar denotes 22Rv1 Scr control cells and the right bar denotes anti-miR-379 22Rv1 cells. Individual values of biological triplicates are shown as blue triangles (scrambled negative control) and orange squares (anti-miR-379). Firefly luciferase normalised to Renilla luciferase signals for measurement. Unpaired two-tailed Student's *t*-tests were performed to compare the treatment groups to one another. Experiments were performed three times and representative data is shown.

**Supplementary Figure S4.** Luciferase reporter vector. Schematic showing important regions, and their sizes, of the pMIR-REPORT luciferase vector used for luciferase reporter assays**.**

**Supplementary Figure S5.** Raw western blots**.** Uncropped blots that were used in Figure 4 are shown. **(a)** is showing the AR blot and **(b)** the β-actinin.

**Supplementary Table S1**. RNA immunoprecipitation of AGO2. From RNA sequencing, the top 500 most significant results were included out of a total of 10411 sequence reads. AR is highlighted in yellow.

**Supplementary Table S2.** Information on TURP patients cohort. Clinical information on patients used in Fig. 7 is shown.
