# Supplementary material for: MicroRNA-379 Modulates Prostate-Specific Antigen Expression Through Targeting the Androgen Receptor in Prostate Cancer
Source: Cancers (Basel). 2025 Oct 7;17(19):3245. doi: 10.3390/cancers17193245 (PMC12524244; doi:10.3390/cancers17193245)
Supplement: Supplementary file 1 [file cancers-17-03245-s001.zip › Supplementary/Supplementary table S2.pdf]

Supplementary Table. 2

		Prostate cancer	Benign prostate hyperplasia
Number of patients		47	23
Age at TURP		75 (63-89) years	69 (56-89) years
WHO grade	Grade I	4	
	Grade II	20	
	Grade III	23	
Clinical stage	T1	9	
	T2	21	
	T3	13	
	T4	3	
	Data missing	1	
PSA at diagnosis		26.7 (0.2-672) ng/ml	6.6 (3-19.4) ng/ml
Metastasis	Yes	25	
	No	8	
	Not suspected	9	
	Data missing	5	
