# Supplementary figures and images for: MicroRNA-379 Modulates Prostate-Specific Antigen Expression Through Targeting the Androgen Receptor in Prostate Cancer
Source: Cancers (Basel). 2025 Oct 7;17(19):3245. doi: 10.3390/cancers17193245 (PMC12524244; doi:10.3390/cancers17193245)

# Supplementary Fig. 1

a

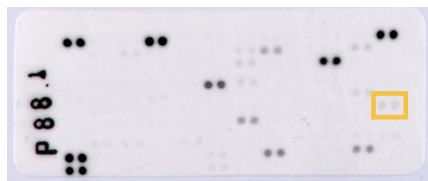

Scr conditioned media

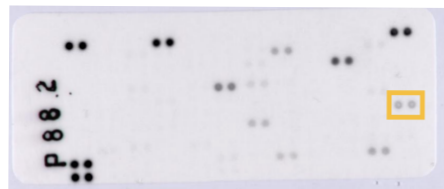

anti-379 conditioned media

b

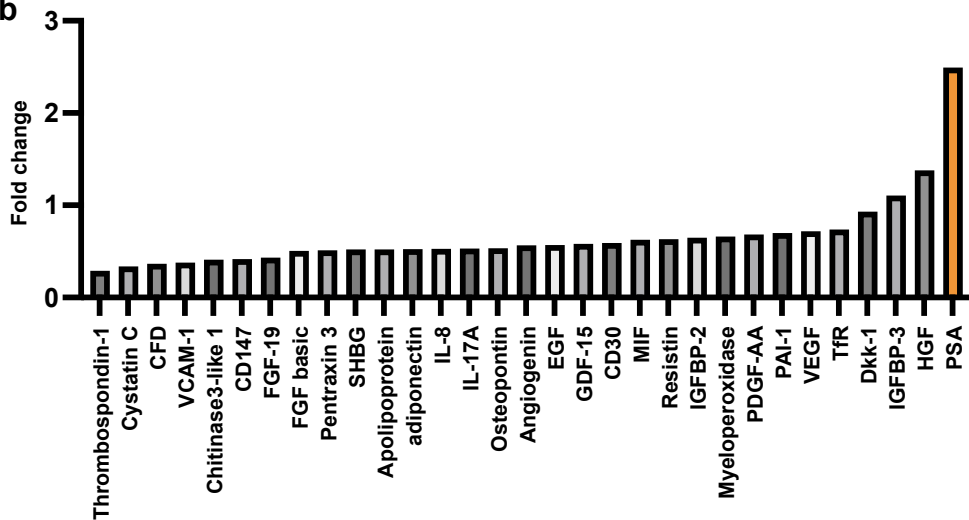

Supplement: Supplementary file 1 [file cancers-17-03245-s001.zip › Supplementary/Supplementary Fig S1.pdf]

# Supplementary Fig. 2

Secreted

Normal medium

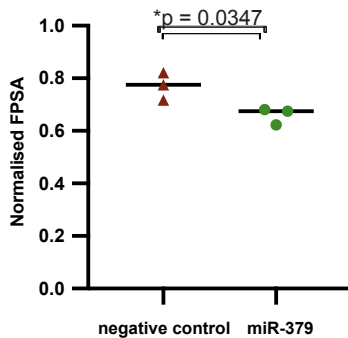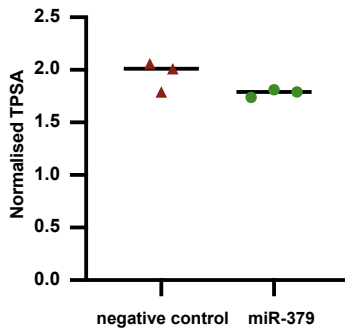

Supplement: Supplementary file 1 [file cancers-17-03245-s001.zip › Supplementary/Supplementary Fig S2.pdf]

**Supplementary Fig. 3**

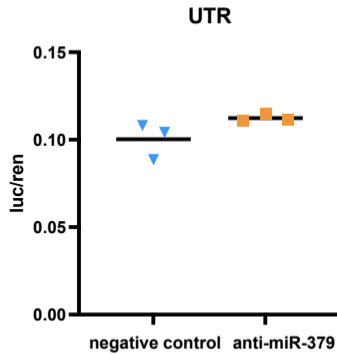

Supplement: Supplementary file 1 [file cancers-17-03245-s001.zip › Supplementary/Supplementary Fig S3.pdf]

# Supplementary Fig. 4

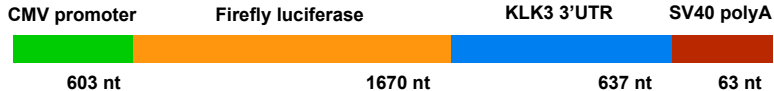

Supplement: Supplementary file 1 [file cancers-17-03245-s001.zip › Supplementary/Supplementary Fig S4.pdf]

# Supplementary Fig. 5

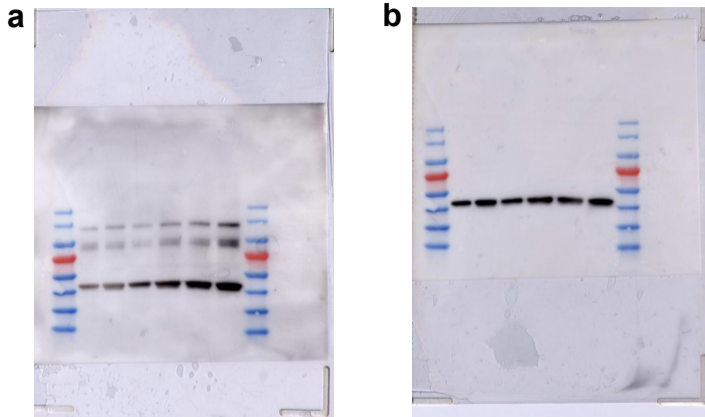

**c**

|                  |   | AR110    | AR95     | Total AR | beta-actin |
|------------------|---|----------|----------|----------|------------|
| miR-379          | 1 | 1132,77  | 4107,66  | 5240,43  | 9532,196   |
|                  | 2 | 2006,669 | 3166,64  | 5173,309 | 12743,68   |
|                  | 3 | 1501,669 | 2096,619 | 3598,288 | 10031,73   |
| Negative control | 4 | 2996,861 | 5446,489 | 8443,35  | 12216,27   |
|                  | 5 | 4690,104 | 6250,267 | 10940,37 | 11194,1    |
|                  | 6 | 5865,083 | 10037,71 | 15902,79 | 14622,46   |

Supplement: Supplementary file 1 [file cancers-17-03245-s001.zip › Supplementary/Supplementary fig S5.pdf]
