# Supplementary material for: MicroRNA-379 Modulates Prostate-Specific Antigen Expression Through Targeting the Androgen Receptor in Prostate Cancer
Source: Cancers (Basel). 2025 Oct 7;17(19):3245. doi: 10.3390/cancers17193245 (PMC12524244; doi:10.3390/cancers17193245)
Supplement: Supplementary file 1 [file Supplementary Table S1.pdf › Supplementary/Supplementary Table S1.pdf]

Supplementary Table. 1

	miR379_1	miR379_2	miR379_3	Control_1	Control_2	Control_3	miR-379 average	C average	t-test
SUPT7L	6.790137088	6.340620171	5.755880423	7.733124486	7.250136145	6.658346557	6.295545894	7.213869063	0.000185196
KIAA1755	5.193475644	5.193475644	5.193475644	6.302942904	6.231495476	6.27800287	5.193475644	6.27081375	0.00037743
SLC29A1	6.425901529	6.340620171	7.310206594	5.193475644	5.193475644	6.10435706	6.692242765	5.497102783	0.000444095
LMNTD2	6.425901114	7.408923746	6.474542398	5.193475644	6.231495476	5.193475644	6.769789086	5.539482255	0.000591552
DDX11	7.529431898	7.408920724	7.721367876	9.298418948	9.263960271	9.411006368	7.553240166	9.324461862	0.000726296
DDX3X	6.425899664	6.340620558	6.474542398	5.193475644	5.193475644	5.193475644	6.41368754	5.193475644	0.001027265
CDC42BPG	6.425901529	6.340620171	6.474542398	5.193475644	5.193475644	5.193475644	6.413688033	5.193475644	0.001027275
RNF167	6.425901529	6.340620171	6.474542398	5.193475644	5.193475644	5.193475644	6.413688033	5.193475644	0.001027275
ACTR3	6.425902772	6.340620171	6.474542398	5.193475644	5.193475644	5.193475644	6.413688447	5.193475644	0.001027278
PLEKHA6	7.572227652	6.340620171	6.474542398	6.376343784	5.193475644	5.193475644	6.79579674	5.587765024	0.00104779
ZNF736	6.473919448	6.340620171	6.474542398	5.193475644	5.193475644	5.193475644	6.429694006	5.193475644	0.001295426
EIF4G2	7.322443252	6.340620171	7.142215542	6.302942904	5.193475644	6.10435706	6.935092988	5.866925203	0.001388375
TRIM17	5.937472053	5.193475644	5.193475644	6.9226234	6.231494973	6.10435739	5.441474447	6.419491921	0.001418507
NAPSA	5.937471983	5.193475644	5.193475644	6.9226234	6.231495476	6.10435706	5.441474424	6.419491979	0.001418525
POLR3H	8.753815552	8.441897719	9.036294618	9.480541236	9.26396208	9.771511043	8.74400263	9.50533812	0.001597193
PAQR4	5.193475644	5.193475644	6.159505402	7.354712658	7.250136145	8.033708627	5.515485564	7.54618581	0.001701424
MLLT6	8.001165918	7.680590095	8.040224354	5.193475644	5.193475644	5.193475644	7.907326789	5.193475644	0.001757681
UBA2	6.425901529	6.617238377	6.488733396	5.193475644	5.193475644	5.193475644	6.510624434	5.193475644	0.001822577
TMEM102	5.937471983	5.193475644	5.193475644	7.354711538	6.828911127	6.658346557	5.441474424	6.947323074	0.001929174
GSK3A	5.937471983	6.971474765	6.952053166	5.193475644	6.231495476	6.10435706	6.620333305	5.843109393	0.002051236
EMC1	6.790137088	7.744131107	7.564913294	7.381212318	8.259366931	8.083379832	7.36639383	7.90798636	0.002083336
NDUFA4	5.937471983	5.193475644	5.755880423	6.841888774	6.231495476	6.658346557	5.628942683	6.577243602	0.002230638
TYW5	5.193475644	5.193475644	5.81675887	6.359576691	6.245584742	7.057644574	5.40123672	6.554268669	0.002258185
GLMP	6.425901529	6.340620171	7.14221572	5.193475644	5.193475644	6.10435706	6.636245807	5.497102783	0.002434509
RALY	6.425901529	6.340620171	7.142215542	5.193475644	5.193475644	6.10435706	6.636245747	5.497102783	0.002434514
GADD45GIP1	6.790137088	7.408922237	7.942096618	8.183696709	8.583134117	9.190162255	7.380385314	8.652331027	0.002556549
CDKL1	7.682974043	6.340620171	6.474542398	6.302942904	5.193475644	5.193475644	6.832712204	5.563298064	0.002813939
ZBTB4	7.871202567	6.340619936	7.7213684	6.922625553	5.193475644	6.658346557	7.311063634	6.258149251	0.002973516
PPP3CA	8.204578678	7.408922237	8.937092457	6.302942904	5.193475644	6.658346557	8.183531124	6.051588368	0.002977559
AARSD1	5.193475644	5.193475644	5.193475644	6.302942904	6.231495476	6.10435706	5.193475644	6.212931813	0.003229304
KLF1	5.193475644	5.193475644	5.193475644	6.302942904	6.231495476	6.10435706	5.193475644	6.212931813	0.003229304
POLR1B	5.193475644	5.193475644	5.193475644	6.302942904	6.231495476	6.10435706	5.193475644	6.212931813	0.003229304
PRIM1	5.193475644	5.193475644	5.193475644	6.302942904	6.231495476	6.10435706	5.193475644	6.212931813	0.003229304
SLC5A4	5.193475644	5.193475644	5.193475644	6.302942904	6.231495476	6.10435706	5.193475644	6.212931813	0.003229304
TEX49	5.193475644	5.193475644	5.193475644	6.302942904	6.231495476	6.10435706	5.193475644	6.212931813	0.003229304
USP45	5.193475644	5.193475644	5.193475644	6.302942904	6.231495476	6.10435706	5.193475644	6.212931813	0.003229304
PSMD4	5.193475644	5.193475644	5.193475644	6.302942742	6.231495124	6.104356846	5.193475644	6.212931571	0.003229306
SCIMP	6.373796557	5.193475644	6.473268277	7.308820784	6.231495476	7.617627717	6.013513493	7.052647992	0.003365118
CCDC78	5.193475644	5.193475644	5.755880423	6.302942904	6.231495476	6.658346557	5.380943904	6.397594979	0.003546273
LEPROTL1	5.193475644	5.193475644	5.755880423	6.302942904	6.231495476	6.658346557	5.380943904	6.397594979	0.003546273
FADS2	5.193475644	5.193475644	6.159505402	6.302942904	6.231495476	7.057644574	5.515485564	6.530694318	0.003716356
CFL1	5.193475644	5.193475644	6.474542398	6.302942904	6.231495476	7.370045892	5.620497896	6.634828091	0.003822343
BACE2	5.193475644	5.193475644	5.193475644	6.320277826	6.26374885	6.10435706	5.193475644	6.229461245	0.00387118
SHROOM3	5.193475644	5.193475644	5.193475644	6.302942904	6.468440877	6.226278947	5.193475644	6.332554243	0.003912239
CLPB	5.937471983	5.193475644	6.159505402	6.9226234	6.231495476	7.370045892	5.763484343	6.841388256	0.003962152
SPDYE5	5.193475644	5.193475644	6.523963414	7.259979524	7.317179928	8.248235688	5.636971568	7.608465047	0.003977453
SPATA2	7.322443252	6.971474765	6.952053166	5.193475644	5.193475644	5.193475644	7.081990394	5.193475644	0.004037071
MAZ	7.322443252	8.015951925	7.835949747	5.193475644	6.231495476	6.10435706	7.724781641	5.843109393	0.004355169
PRC1	5.193475644	5.193475644	5.755880423	6.302942904	6.231495476	7.057644574	5.380943904	6.530694318	0.004659205
SLCO1A2	7.598970848	7.744131107	7.521994473	8.198913702	8.4868212	8.135560917	7.621698809	8.273765273	0.004829956
PNPO	6.707178775	6.605221282	6.382209169	5.193475644	5.193475644	5.193475644	6.564869742	5.193475644	0.004860065
ATP6AP1	6.425901529	6.340620171	6.159505402	5.193475644	5.193475644	5.193475644	6.308675701	5.193475644	0.004923726
IPO13	6.425901529	6.340620171	6.159505402	5.193475644	5.193475644	5.193475644	6.308675701	5.193475644	0.004923726
PANX2	6.425901529	6.340620171	6.159505402	5.193475644	5.193475644	5.193475644	6.308675701	5.193475644	0.004923726
PREB	6.425901529	6.340620171	6.159505402	5.193475644	5.193475644	5.193475644	6.308675701	5.193475644	0.004923726
SPATA20	6.425901529	6.340620171	6.159505402	5.193475644	5.193475644	5.193475644	6.308675701	5.193475644	0.004923726
UBE2Q1	6.425901529	6.340620171	6.159505402	5.193475644	5.193475644	5.193475644	6.308675701	5.193475644	0.004923726
ZBTB39	6.425901529	6.340620171	6.159505402	5.193475644	5.193475644	5.193475644	6.308675701	5.193475644	0.004923726
C9orf131	5.193475644	6.340620195	6.952053214	6.30294295	7.250136814	7.84450738	6.162049685	7.132529048	0.00511395
RPSA	7.710413888	6.971474765	6.952053166	6.302942904	5.193475644	5.193475644	7.21131394	5.563298064	0.005295316
TAPBP	6.435976194	7.021286973	6.989090166	7.289251736	8.096276859	8.08380464	6.815451111	7.823111078	0.005848608
CAPN15	6.790137088	6.971474765	7.46066066	5.193475644	5.193475644	6.10435706	7.074090838	5.497102783	0.005944298
SLX1B	5.612911372	5.193475644	5.193475644	6.302942904	5.803895699	5.719641561	5.333287554	5.942160055	0.005983378
SLC9A4	6.074917302	6.340620171	6.162020584	5.193475644	5.193475644	5.193475644	6.192519352	5.193475644	0.006071676
HS3ST1	6.21165284	6.450341508	6.178258028	5.193475644	5.193475644	5.193475644	6.280084125	5.193475644	0.00615904
TMED2	6.425901529	6.340620171	7.596895559	5.193475644	5.193475644	6.658346557	6.787805753	5.681765949	0.006170821
SLC15A2	5.193475644	5.193475644	5.193475644	6.390924188	6.231495476	6.10435706	5.193475644	6.242258908	0.00618995
TLNRD1	5.937471983	6.971474765	7.596895559	5.193475644	6.231495476	6.658346557	6.835280769	6.027772559	0.006521248
TECRL	5.193475644	6.340620171	6.159505402	6.302942904	7.250136145	7.370045892	5.897867073	6.97437498	0.00668286
DLX1	8.982303922	8.910193364	9.29854814	9.642236038	9.724644056	9.922790306	9.063681808	9.763223467	0.00689064
BCL7B	6.425901529	7.195783232	6.159505402	5.193475644	6.231495476	5.193475644	6.593730054	5.539482255	0.007065698
ZFAND5	6.425901529	7.408922237	6.732964383	5.193475644	6.231495476	5.193475644	6.855929383	5.539482255	0.007242381
MOB3A	5.959084271	6.340620171	6.25812469	6.382905632	6.892120514	6.695400178	6.185943044	6.656808775	0.00731834
INPP5A	7.322443252	6.340620171	6.952053166	6.302942904	5.193475644	6.10435706	6.87170553	5.866925203	0.007372823
LTB4R2	5.937471983	5.193475644	5.755880423	6.9226234	6.231495476	7.057644574	5.628942683	6.737254484	0.00771604
CAV2	5.937471983	5.193475644	6.059700091	7.354711538	6.231495476	7.354354487	5.730215906	6.980187167	0.007895067
CEP41	6.425901529	6.340620171	6.098782365	5.193475644	5.193475644	5.193475644	6.288434688	5.193475644	0.007910633
SLC26A6	5.937471983	5.193475644	5.755880423	7.35471203	6.231495476	7.057644574	5.628942683	6.881284027	0.007935009
HIST1H3H	6.425901529	6.340620171	6.732964383	5.193475644	5.193475644	5.193475644	6.499828694	5.193475644	0.008215055
PKN3	5.937471983	6.971474765	6.159505402	5.193475644	6.231495476	5.193475644	6.356150717	5.539482255	0.008260794
GSTO2	6.425901529	6.340620171	6.474542398	7.956525514	8.06540678	8.562595809	6.413688033	8.194842701	0.008307672
PABPC4	7.322443252	6.971474765	6.732964383	5.193475644	5.193475644	5.193475644	7.0089608	5.193475644	0.008775277
OLA1	5.971905872	5.193475644	5.193475644	7.445688455	6.291333223	6.689757735	5.452952387	6.808926471	0.008959832
ACOT11	8.905505004	8.896240628	9.034164068	9.642236038	9.434394741	9.620701387	8.945303233	9.565777389	0.009156081
TNFRSF10B	6.790137088	7.408922237	7.310206594	5.193475644	5.193475644	5.193475644	7.169755306	5.193475644	0.009300913
SMIM19	6.425901529	6.340620171	6.159505402	7.68677412	7.250136145	7.376606642	6.308675701	7.437838969	0.009449301
ACOT9	5.193475644	5.193475644	5.193475644	6.515799376	6.231495476	6.658346557	5.193475644	6.468547136	0.009543987
EIF4G1	7.322443007	7.744131107	9.036292465	5.193475644	6.231495817	7.057643116	8.03428886	6.160871526	0.009666249
DPYSL2	6.790137088	6.340620171	6.474542398	5.193475644	5.193475644	5.193475644	6.535099886	5.193475644	0.009720858
TWIST1	6.790137088	6.340620171	6.474542398	5.193475644	5.193475644	5.193475644	6.535099886	5.193475644	0.009720858
ZNF471	8.695307414	8.203289638	8.905657449	9.711175855	9.001123051	9.637279911	8.601418167	9.449859606	0.01008802
L2HGDH	6.733041548	6.642922054	7.710419958	7.781880388	8.036729797	8.73407514	7.02879452	8.184228441	0.010512097
H2AFZ	7.080702557	7.408922237	6.732964383	5.193475644	5.193475644	5.193475644	7.074196392	5.193475644	0.010597019
PRPS1	5.193475644	5.193475644	6.159505402	6.9226234	6.828911812	7.370045892	5.515485564	7.040527035	0.01077027
NEPRO	7.322443252	6.971474765	6.474542398	8.379915508	8.259366931	7.370045892	6.922820138	8.003109444	0.010924273
VIM	7.322443252	6.340620171	7.7213684	8.183696709	7.575763645	8.724835423	7.128143941	8.161431926	0.010937124
C16orf54	6.425901529	6.340620171	6.048842156	5.193475644	5.193475644	5.193475644	6.271787952	5.193475644	0.011023104
GNA11	6.790137088	6.971474765	7.46066066	5.193475644	5.193475644	5.193475644	7.074090838	5.193475644	0.011148769
SNTB2	6.790137088	6.340620171	6.821819465	5.193475644	5.193475644	5.193475644	6.650858908	5.193475644	0.011177946
TMEM106A	6.425901529	6.461809303	6.732964383	7.251072994	7.575763645	7.541609333	6.540225072	7.456148657	0.011511816
NUDT19	5.193475644	5.193475644	5.193475644	6.329304888	6.857463696	6.660681601	5.193475644	6.615816728	0.011536523
KLHL23	5.193475644	5.193475644	5.193475644	6.465586178	6.828911812	7.057644574	5.193475644	6.784047521	0.011542204
NSD1	6.425901529	6.340620171	6.952053166	5.193475644	5.193475644	6.10435706	6.572858289	5.497102783	0.011556195
PHB	6.425901529	6.340620171	6.952053166	5.193475644	5.193475644	6.10435706	6.572858289	5.497102783	0.011556195
HEATR5A	6.4629971	6.971474765	6.535756859	5.193475644	5.193475644	5.193475644	6.656742908	5.193475644	0.011567932
UQCRC1	6.425901529	7.744131107	7.142215542	5.193475644	6.231495476	6.10435706	7.104082726	5.843109393	0.011732109
CADM4	5.193475644	5.193475644	5.193475644	6.302942904	6.231495476	6.658346557	5.193475644	6.397594979	0.011804472
CCT8	5.193475644	5.193475644	5.193475644	6.302942904	6.231495476	6.658346557	5.193475644	6.397594979	0.011804472
P2RY8	5.193475644	5.193475644	5.193475644	6.302942904	6.231495476	6.658346557	5.193475644	6.397594979	0.011804472
CASC3	6.510566039	6.971474765	6.474542398	5.193475644	5.193475644	5.193475644	6.652194401	5.193475644	0.0118149
C1orf21	5.937471983	6.340620171	5.755880423	6.302942904	6.828911812	6.10435706	6.011324192	6.412070592	0.011865964
DDX19A	5.937471983	6.340620171	5.755880423	6.302942904	6.828913573	6.10435706	6.011324192	6.412071179	0.011866239
FLNA	6.425901529	6.971472434	7.835951949	5.193475644	5.193475644	6.104356791	7.077775304	5.497102693	0.011987498
POGZ	6.790138375	6.971474765	7.310206594	5.193475644	5.193475644	6.104357171	7.023939911	5.49710282	0.012004968
BAZ1A	7.080702557	6.340619785	6.159504885	6.302942872	5.193475644	5.193475644	6.526942409	5.563298054	0.012025615
CEP68	5.937471983	5.193475644	5.193475644	7.460556278	7.250136145	6.667257295	5.441474424	7.125983239	0.012052022
EIF2AK1	7.871202567	8.441897719	8.133492282	8.552613963	8.963341285	8.610603408	8.148864189	8.708852885	0.012054632
SP2	6.425901529	6.971474765	6.952053166	5.193475644	5.193475644	5.193475644	6.783143153	5.193475644	0.012403382
TRPS1	7.52943113	7.744131107	8.040966072	8.183696709	8.259366931	8.486542743	7.771509436	8.309868795	0.012735699
B3GALT2	6.425901529	5.193475644	5.193475644	7.956525514	6.231495476	6.658346557	5.604284272	6.948789182	0.01293469
NOCT	5.193475644	5.193475644	5.193475644	6.9226234	6.34437996	6.658346557	5.193475644	6.641783306	0.013056161
CTDSP1	6.790137088	7.408922237	7.596895559	5.193475644	5.193475644	5.193475644	7.265318295	5.193475644	0.013555832
FBP2	6.425901529	6.340620171	7.142215542	7.68677412	7.250136145	8.033708627	6.636245747	7.656872964	0.013595713
CBY1	6.790137088	6.971474765	7.310206594	6.302942904	6.231495476	6.658346557	7.023939482	6.397594979	0.013701647
ZFP36L1	7.080702557	7.744132303	6.952053166	5.193475644	5.193475644	5.193475644	7.258962675	5.193475644	0.013824963
FAM120A	6.790136445	6.971473232	7.543664061	5.193475644	5.193475644	5.193475644	7.101757912	5.193475644	0.013865191
SNED1	8.37897825	6.971474765	8.937092457	9.298418948	7.575763645	9.771511043	8.095848491	8.881897878	0.014040769
BCL9L	6.790137088	7.408922237	6.732964383	5.193475644	5.193475644	5.193475644	6.977341236	5.193475644	0.014401642
MRPL48	5.937471983	5.193475644	5.755880423	7.211275485	6.279820262	6.584343878	5.628942683	6.691813208	0.014433247
RBM8A	6.425901529	5.193475644	5.193475644	7.100957373	6.231495476	6.10435706	5.604284272	6.478936636	0.014459895
TMEM54	6.425901529	6.971474765	6.474542398	5.193475644	5.193475644	5.193475644	6.623972897	5.193475644	0.01452657
OCRL	6.425902896	6.971476042	6.474542398	5.193475644	5.193475644	5.193475644	6.623973779	5.193475644	0.014526579
SULT2A1	5.937471983	6.340620171	6.197744108	5.193475644	5.193475644	5.193475644	6.158612087	5.193475644	0.014624034
ADSSL1	5.937471983	6.340620171	6.159505402	5.193475644	5.193475644	5.193475644	6.145865852	5.193475644	0.014654734
CD151	5.937471983	6.340620171	6.159505402	5.193475644	5.193475644	5.193475644	6.145865852	5.193475644	0.014654734
FAM72A	5.937471983	6.340620171	6.159505402	5.193475644	5.193475644	5.193475644	6.145865852	5.193475644	0.014654734
FBXL14	5.937471983	6.340620171	6.159505402	5.193475644	5.193475644	5.193475644	6.145865852	5.193475644	0.014654734
GET4	5.937471983	6.340620171	6.159505402	5.193475644	5.193475644	5.193475644	6.145865852	5.193475644	0.014654734
GLRA1	5.937471983	6.340620171	6.159505402	5.193475644	5.193475644	5.193475644	6.145865852	5.193475644	0.014654734
KLF3	5.937471983	6.340620171	6.159505402	5.193475644	5.193475644	5.193475644	6.145865852	5.193475644	0.014654734
MRPL9	5.937471983	6.340620171	6.159505402	5.193475644	5.193475644	5.193475644	6.145865852	5.193475644	0.014654734
MYL12A	5.937471983	6.340620171	6.159505402	5.193475644	5.193475644	5.193475644	6.145865852	5.193475644	0.014654734
NACC2	5.937471983	6.340620171	6.159505402	5.193475644	5.193475644	5.193475644	6.145865852	5.193475644	0.014654734
NXPH4	5.937471983	6.340620171	6.159505402	5.193475644	5.193475644	5.193475644	6.145865852	5.193475644	0.014654734
RAMP1	5.937471983	6.340620171	6.159505402	5.193475644	5.193475644	5.193475644	6.145865852	5.193475644	0.014654734
RPTOR	5.937471983	6.340620171	6.159505402	5.193475644	5.193475644	5.193475644	6.145865852	5.193475644	0.014654734
RRP9	5.937471983	6.340620171	6.159505402	5.193475644	5.193475644	5.193475644	6.145865852	5.193475644	0.014654734
SENP3	5.937471983	6.340620171	6.159505402	5.193475644	5.193475644	5.193475644	6.145865852	5.193475644	0.014654734
TEPSIN	5.937471983	6.340620171	6.159505402	5.193475644	5.193475644	5.193475644	6.145865852	5.193475644	0.014654734
USP11	5.937471983	6.340620171	6.159505402	5.193475644	5.193475644	5.193475644	6.145865852	5.193475644	0.014654734
XPO5	5.937471983	6.340620171	6.159505402	5.193475644	5.193475644	5.193475644	6.145865852	5.193475644	0.014654734
ZDHHC9	5.937471983	6.340620171	6.159505402	5.193475644	5.193475644	5.193475644	6.145865852	5.193475644	0.014654734
ZNF672	5.937471983	6.340620171	6.159505402	5.193475644	5.193475644	5.193475644	6.145865852	5.193475644	0.014654734
CASC4	5.937471983	6.340620171	6.216337678	5.193475644	5.193475644	5.193475644	6.164809944	5.193475644	0.01472695
PRMT6	7.52943113	6.340620171	7.46066066	6.302942904	5.193475644	6.658346557	7.11023732	6.051588368	0.014790578
DUSP12	5.937471983	6.340620171	6.159505402	7.354711538	7.250136145	7.370045892	6.145865852	7.324964525	0.015272232
IL17RB	5.937471983	6.340620171	6.159505402	7.354711538	7.250136145	7.370045892	6.145865852	7.324964525	0.015272232
RNH1	5.937471983	5.193475644	5.193475644	7.354714525	7.250136145	7.370045892	5.441474424	7.324965521	0.01529898
RBBP8	7.322443252	6.340620171	6.952053166	8.379915508	7.250137073	7.626692885	6.87170553	7.752248489	0.01564758
ARPC5	9.129222325	8.334805951	7.347441584	7.642342754	7.329063917	6.279857939	8.270489953	7.08375487	0.015833778
RPAP2	10.67262203	10.08683757	10.87000209	11.80763756	11.3042883	11.65304777	10.5431539	11.58832454	0.015857767
IBA57	7.898470672	6.630075863	6.733872639	8.648838815	7.298154611	7.20827889	7.087473058	7.718424105	0.016394841
RYK	5.937471983	7.408922614	6.159507163	5.193475644	6.231495476	5.193475644	6.501967253	5.539482255	0.01648593
PRRG4	6.555643512	6.340620171	6.474542398	7.049268362	7.125692144	7.168798909	6.456935361	7.114586472	0.016712224
AGO2	5.937471983	6.340620171	6.952053166	5.193475644	5.193475644	6.10435706	6.41004844	5.497102783	0.017079483
FKBP4	5.937471983	6.340620171	6.952053166	5.193475644	5.193475644	6.10435706	6.41004844	5.497102783	0.017079483
RPS10	5.937471983	6.340620171	6.952053166	5.193475644	5.193475644	6.10435706	6.41004844	5.497102783	0.017079483
PPP1CC	7.52943113	6.971474765	7.596895559	5.193475644	5.193475644	6.10435706	7.365933818	5.497102783	0.017114176
C15orf40	8.163058955	8.133762689	8.977375505	9.141466022	9.488334875	9.862521845	8.424732383	9.497440914	0.017424206
UGDH	6.568248674	7.190128021	6.732964383	5.193475644	6.231495476	5.193475644	6.830447026	5.539482255	0.017456143
AHCYL1	6.790137088	6.340620171	7.142215542	5.193475644	5.193475644	6.10435706	6.7576576	5.497102783	0.017907029
MYH7B	5.937471983	5.193475644	5.755880423	7.354711538	6.231495476	6.658346557	5.628942683	6.748184524	0.018422668
DDX17	6.425903186	6.340620171	6.952053166	5.193475644	5.193475644	5.193475644	6.572858841	5.193475644	0.01867478
SLC9A1	6.425901529	6.340620171	6.952053166	5.193475644	5.193475644	5.193475644	6.572858289	5.193475644	0.018674835
POMK	5.937471983	6.340620171	7.565970905	5.193475644	5.193475644	6.758179274	6.614687686	5.715043521	0.018796726
SSBP2	6.734853494	6.971474765	6.248759237	8.090721424	7.830626439	7.611092306	6.651695832	7.844146723	0.018979266
SRCIN1	7.080702557	6.340620171	6.732964383	5.193475644	5.193475644	5.193475644	6.718095704	5.193475644	0.019098494
PTEN	9.761342785	9.708577374	10.23528005	9.642235273	9.512613997	10.05970046	9.901733404	9.738183245	0.019187974
CPOX	5.937471983	6.340620171	7.310206594	5.193475644	5.193475644	6.10435706	6.529432916	5.497102783	0.019204417
CSNK1D	7.080702557	6.340620171	6.474541832	6.302942904	5.193475644	5.193475644	6.631954853	5.563298064	0.019261863
PYGO2	6.790137088	7.408922237	7.142215542	5.193475644	6.231495476	5.193475644	7.113758289	5.539482255	0.019470822
TMEM190	5.193475644	5.193475644	5.193475644	6.9226234	6.231495476	6.658346557	5.193475644	6.604155144	0.019769113
R3HDM2	5.937471983	5.193475644	5.995234248	6.9226234	6.82891183	7.45407126	5.708727292	7.068535497	0.019780804
RBL2	5.937471983	5.193475644	5.193475644	6.9226234	6.828911812	6.658346557	5.441474424	6.803293923	0.01982734
CDHR5	7.322443252	6.971474765	5.755880423	8.379915508	8.06540678	7.370045892	6.683266147	7.93845606	0.01990766
OR7D2	6.444869134	6.409025611	5.96267099	5.193475644	5.193475644	5.193475644	6.272188578	5.193475644	0.020054712
HYAL1	5.193475644	5.193475644	5.755880423	6.302942904	6.231495476	7.370044768	5.380943904	6.634827716	0.020276623
ATR	7.710413888	7.408922237	7.942096618	8.183696709	8.06540678	8.350801506	7.687144248	8.199968332	0.020304875
PNPLA6	6.790137088	6.971474765	7.721369124	5.193475644	5.193475644	5.193475644	7.160993659	5.193475644	0.02034912
PLEC	7.52943113	8.015951925	8.302444778	6.9226234	7.250134581	7.844507139	7.949275944	7.339088374	0.02057062
SPRN	7.080702557	7.408922237	7.835949747	7.68677412	8.430313939	8.724835423	7.44185818	8.280641161	0.020664176
NDUFA9	6.442515872	5.193475644	5.838794044	7.952983205	7.729103485	8.149609803	5.82492852	7.943898831	0.020881776
VPS26C	5.193475644	5.193475644	6.187006671	6.302942904	6.828911812	7.235723037	5.524652653	6.789192584	0.02101765
USP22	7.080702557	6.340620171	6.511123891	6.302942904	5.193475644	5.193475644	6.644148873	5.563298064	0.021048726
COX18	8.040529969	7.408922237	7.185601939	8.744135812	7.895919506	7.626693983	7.545018048	8.088916433	0.021439245
TUBGCP5	5.937471983	5.193475644	6.159505402	6.922624408	6.828911812	7.370045892	5.763484343	7.040527371	0.021567549
ZNF667	6.425901529	5.193475644	6.474542398	7.558888104	6.231495476	7.144021335	6.031306524	6.978134972	0.021570979
GCN1	6.790137088	7.744131107	6.952053166	5.193475644	5.193475644	5.193475644	7.162107121	5.193475644	0.021689209
ELL3	5.937471983	5.193475644	5.755880423	6.9226234	6.828911812	7.370045892	5.628942683	7.040527035	0.022080822
DOCK7	6.790137088	6.340620171	6.159507163	5.193475644	5.193475644	5.193475644	6.430088141	5.193475644	0.022217687
JMY	6.790137088	6.340620171	6.159505402	5.193475644	5.193475644	5.193475644	6.430087554	5.193475644	0.022217804
PGBD2	6.790137088	6.340620171	6.159505402	5.193475644	5.193475644	5.193475644	6.430087554	5.193475644	0.022217804
HNRNPU	7.384397884	7.408922237	7.835949747	6.9226234	6.828911812	7.057644574	7.543089956	6.936393262	0.022391851
ACACA	5.937472541	6.340620171	6.474542398	5.193475644	5.193475644	5.193475644	6.25087837	5.193475644	0.02251493
ACACB	5.937471983	6.340620171	6.474542398	5.193475644	5.193475644	5.193475644	6.250878184	5.193475644	0.022514986
AHSA1	5.937471983	6.340620171	6.474542398	5.193475644	5.193475644	5.193475644	6.250878184	5.193475644	0.022514986
ATP6V1B2	5.937471983	6.340620171	6.474542398	5.193475644	5.193475644	5.193475644	6.250878184	5.193475644	0.022514986
B3GNTL1	5.937471983	6.340620171	6.474542398	5.193475644	5.193475644	5.193475644	6.250878184	5.193475644	0.022514986
CERS5	5.937471983	6.340620171	6.474542398	5.193475644	5.193475644	5.193475644	6.250878184	5.193475644	0.022514986
FOXK2	5.937471983	6.340620171	6.474542398	5.193475644	5.193475644	5.193475644	6.250878184	5.193475644	0.022514986
MGAT4B	5.937471983	6.340620171	6.474542398	5.193475644	5.193475644	5.193475644	6.250878184	5.193475644	0.022514986
RNF220	5.937471983	6.340620171	6.474542398	5.193475644	5.193475644	5.193475644	6.250878184	5.193475644	0.022514986
SLC12A7	5.937471983	6.340620171	6.474542398	5.193475644	5.193475644	5.193475644	6.250878184	5.193475644	0.022514986
TFRC	5.937471983	6.340620171	6.474542398	5.193475644	5.193475644	5.193475644	6.250878184	5.193475644	0.022514986
WDPCP	5.937471983	6.340620171	6.474542398	5.193475644	5.193475644	5.193475644	6.250878184	5.193475644	0.022514986
WFIKKN1	5.937471983	6.340620171	6.474542398	5.193475644	5.193475644	5.193475644	6.250878184	5.193475644	0.022514986
ZFYVE21	5.937471983	6.340620171	6.474542398	5.193475644	5.193475644	5.193475644	6.250878184	5.193475644	0.022514986
NACA	6.425901529	6.971474765	6.952053166	6.302942904	6.828911812	6.749054254	6.783143153	6.626969657	0.022971093
FLG	10.03480261	10.46614394	10.71114489	10.72169448	11.39677676	11.25987786	10.40403048	11.12611637	0.023079451
DHX15	7.080702557	6.340620171	7.46066066	6.302942904	5.193475644	6.10435706	6.960661129	5.866925203	0.023087875
A1CF	5.193475644	5.193475644	5.193475644	6.9226234	6.272020482	7.05764425	5.193475644	6.750762711	0.023405193
MED14OS	6.790137088	8.244587753	6.952053166	6.302942904	7.575763645	6.10435706	7.328926003	6.661021203	0.023428205
DCAF1	9.23966988	9.78325528	9.934583852	9.563654765	10.26625147	10.50557913	9.652503004	10.11182846	0.023879987
AEBP2	6.925051395	6.971474765	7.142215542	5.193475644	5.193475644	6.10435706	7.012913901	5.497102783	0.024038109
ORMDL2	7.784038593	8.015951925	8.348997783	8.183696709	8.72130582	8.92929174	8.049662767	8.611431423	0.024051885
ZNF7	5.193475644	5.193475644	5.755880423	6.442829129	6.828911812	6.694646734	5.380943904	6.655462558	0.024096561
CLPP	6.790137088	6.340620171	7.596895559	5.193475644	5.193475644	6.658346557	6.909217606	5.681765949	0.024124006
CERKL	5.937471983	6.340620171	5.755880423	7.354711538	7.250136145	6.658346557	6.011324192	7.087731413	0.024163692
IL17RA	6.096781992	6.340620144	5.193475644	8.03715531	7.575762689	7.370046361	5.87695926	7.66098812	0.024216473
NEDD9	7.128644831	6.360869004	6.515660165	5.193475644	5.193475644	5.193475644	6.668391333	5.193475644	0.024343627
TNS2	5.937471983	6.340620171	7.46066066	8.183696709	8.259366931	8.724835423	6.579584271	8.389299688	0.02451505
TXNIP	8.267791991	8.015951925	9.29854814	6.302942904	5.193475644	7.626693983	8.527430685	6.374370844	0.02475924
TIGD5	5.937471983	5.193475644	6.482242406	7.354711538	7.095032774	7.568702398	5.871063344	7.339482237	0.025009765
AGO3	7.826004913	7.942678941	7.47144302	8.635017602	8.481299847	7.961566965	7.746708958	8.359294805	0.025238072
FNTB	6.397401084	6.340620171	5.755880423	6.9226234	7.250136145	6.658346557	6.164633893	6.943702034	0.025536018
FBXO11	7.080702557	7.408922237	7.310206594	5.193475644	6.231495476	6.10435706	7.266610463	5.843109393	0.025550146
RASGRP4	5.193475644	5.193475644	5.193475644	6.986920808	6.828911812	6.1964229	5.193475644	6.67075184	0.025701111
ZNF706	7.080702557	6.97147221	7.310206594	6.302942904	6.231495476	6.10435706	7.120793787	6.212931813	0.026025632
HNRNPA2B1	6.790137318	7.408922237	7.310206594	5.193475644	6.231495476	5.193475644	7.169755306	5.539482255	0.026664289
FLCN	8.075318319	8.647652975	7.896021512	7.68677412	7.956446363	7.39133972	8.206330935	7.678186734	0.026747834
BAZ2A	7.322443252	7.744131412	7.310206594	5.193475644	6.231495574	6.10435706	7.458927086	5.843109426	0.027077302
F2RL1	5.937471983	6.521754222	6.159505402	5.193475644	5.193475644	5.193475644	6.206243869	5.193475644	0.027123605
PRR12	7.322443252	6.340620171	7.835949747	6.302942904	5.193475644	6.10435706	7.166337723	5.866925203	0.027299254
DGKQ	6.425901529	5.193475644	6.474542398	7.68677412	6.828911812	7.370045892	6.031306524	7.295243941	0.027392939
UPF1	7.529430937	6.971475685	6.474542398	5.193475644	5.193475644	5.193475644	6.99181634	5.193475644	0.027526225
INPP5F	6.425901529	7.408922237	6.474542398	6.9226234	8.06540678	7.370045892	6.769788721	7.452692024	0.027604103
NUTF2	7.322443252	6.971474765	6.732964146	6.9226234	6.231495476	6.10435706	7.008960721	6.419491979	0.027660558
CALM1	6.790137088	6.971474765	6.159505402	5.193475644	5.193475644	5.193475644	6.640372419	5.193475644	0.02772487
CDV3	6.790137088	6.971476298	6.159505402	5.193475644	5.193475644	5.193475644	6.64037293	5.193475644	0.027724925
HSPE1	7.080702557	6.971474765	7.142215542	5.193475644	5.193475644	6.10435706	7.064797621	5.497102783	0.027760457
DDB1	8.753816377	6.971474765	7.46066066	6.302942904	5.193475644	6.10435706	7.7286506	5.866925203	0.028082248
WDR37	6.036112918	6.340620171	6.732964383	5.193475644	5.193475644	5.193475644	6.369899158	5.193475644	0.028158819
ITGA9	5.937471983	6.971474765	6.474542398	7.956525514	8.06540678	8.033708627	6.461163049	8.018546974	0.028169009
VPS4A	8.753816377	9.052871444	9.310040565	9.860277741	9.693655912	10.04504173	9.038909462	9.866325126	0.028269285
CEBPD	6.425901529	6.971474765	6.159505402	6.9226234	7.250136145	6.658346557	6.518960565	6.943702034	0.028323126
JAK3	5.937471983	6.453791428	6.57216006	5.193475644	5.193475644	5.193475644	6.321141157	5.193475644	0.028583844
FBRS	6.790137088	6.340620171	7.310206594	5.193475644	5.193475644	5.193475644	6.813654618	5.193475644	0.028619999
NANOG	5.937471983	5.193475644	6.4412612	7.139780698	6.231495476	7.081273363	5.857402942	6.817516512	0.028923731
MYEF2	5.937471983	6.340620171	5.859899828	5.193475644	5.193475644	5.193475644	6.045997327	5.193475644	0.029215922
ZKSCAN3	7.476046831	7.408920896	7.33631924	7.237327609	7.250137935	7.200270199	7.407095656	7.229245247	0.029304545
PTBP3	5.937471983	6.557372787	6.159506107	5.193475644	5.193475644	5.193475644	6.218116959	5.193475644	0.029920837
C9orf3	5.193475644	5.193475644	5.193475644	6.302942904	6.828911812	6.10435706	5.193475644	6.412070592	0.030054253
PACS2	5.193475644	5.193475644	5.193475644	6.302942904	6.828911812	6.10435706	5.193475644	6.412070592	0.030054253
PGM5	5.193475644	5.193475644	5.193475644	6.302942904	6.828911812	6.10435706	5.193475644	6.412070592	0.030054253
TSPEAR	5.193475644	5.193475644	5.193475644	6.302942674	6.828913422	6.104357003	5.193475644	6.412071033	0.030054379
ENPP6	5.937471983	6.340620171	6.611247367	5.193475644	5.193475644	5.193475644	6.296446507	5.193475644	0.030084019
RASAL2	6.425901529	6.971474765	6.169938459	5.193475644	5.193475644	5.193475644	6.522438251	5.193475644	0.03020694
BEND3	8.267791991	8.015951925	8.65438883	8.706831987	8.259366931	9.10833687	8.312710915	8.691511929	0.030599849
TRRAP	6.42590099	6.340620401	7.310206895	5.193475644	5.193475644	6.658344671	6.692242762	5.68176532	0.03061583
ARIH2OS	5.937471983	5.193475644	5.964127063	7.354711538	7.532315757	7.370045892	5.69835823	7.419024396	0.030788698
KIN	6.517056991	5.193475644	6.667062973	8.027423084	7.372583906	7.853590446	6.125865203	7.751199145	0.030841875
USF2	6.425901529	6.971474765	6.159505402	5.193475644	5.193475644	5.193475644	6.518960565	5.193475644	0.031000251
AR	9.016150506	7.744131149	9.212293327	6.922623471	6.38049259	8.033708847	8.657524994	7.112274969	0.031150073
NAALAD2	5.193475644	5.193475644	6.474542398	6.302942904	6.231495476	7.057644574	5.620497896	6.530694318	0.031269292
GPR155	6.670613134	7.424871187	6.822707502	8.035341885	8.148503599	7.813896071	6.972730608	7.999247185	0.031269542
EMC3	6.425901529	6.971474765	7.59159743	6.9226234	7.389292538	8.343654413	6.996324575	7.551856784	0.03142197
HDAC1	6.790137088	6.340620171	6.952053166	5.193475644	5.193475644	6.10435706	6.694270142	5.497102783	0.031499246
SLC25A6	7.52943113	7.408922237	8.380037604	6.9226234	6.231495476	7.370045892	7.77279699	6.841388256	0.031503207
ATP8B2	6.790138698	6.340620171	7.504813564	5.193475644	5.193475644	6.658346557	6.878524144	5.681765949	0.031610821
TUBG1	6.46679208	7.408922237	7.7213684	5.193475644	6.231495476	7.057644574	7.199027572	6.160871898	0.031661896
EIF5A	7.871202567	8.244587753	8.518772442	6.302942904	5.193475644	6.10435706	8.211520921	5.866925203	0.031955338
TRABD	5.193475644	5.193475644	5.755880423	6.9226234	6.828911812	6.658346557	5.380943904	6.803293923	0.032142336
EML2	5.193475644	5.193475644	5.755880651	6.922623839	6.828913524	6.658345701	5.38094398	6.803294355	0.032142479
FZD5	5.937471983	6.340620171	6.732964383	5.193475644	5.193475644	6.10435706	6.337018846	5.497102783	0.033283771
GARS	5.937471983	6.340620171	6.732964383	5.193475644	5.193475644	6.10435706	6.337018846	5.497102783	0.033283771
SUCO	5.937471983	6.340620171	6.732964383	5.193475644	5.193475644	6.10435706	6.337018846	5.497102783	0.033283771
HMG20A	7.529431273	6.340620178	6.732963852	6.302943245	5.193475644	6.104357314	6.867671768	5.866925401	0.033348629
AP2A1	6.425901529	6.340620171	6.732964383	5.193475644	5.193475644	6.10435706	6.499828694	5.497102783	0.033628293
GRK6	7.08070203	6.971474765	6.159505402	5.193475644	5.193475644	5.193475644	6.737227399	5.193475644	0.033651475
FKBP5	8.376815032	6.971474765	7.142215542	6.302942904	5.193475644	6.10435706	7.496835113	5.866925203	0.033924337
SLC26A10	5.193475644	5.193475644	5.755880423	7.956525514	6.828911812	7.370045892	5.380943904	7.385161073	0.034028117
RILPL2	9.276555324	9.696710123	9.223014617	9.491174285	10.12116718	9.534611714	9.398760021	9.715651061	0.034714575
MAP4	6.425901529	7.408922237	7.46066066	5.193475644	5.193475644	6.10435706	7.098494808	5.497102783	0.035296476
DRC3	5.937471983	6.340620171	6.474542398	6.9226234	6.828911812	7.370045892	6.250878184	7.040527035	0.03550115
ATN1	7.390529749	8.244586973	7.596895734	6.302943202	7.250137609	7.057644574	7.744004152	6.870241795	0.035593715
RNF6	5.937471983	6.340620171	5.755880423	6.9226234	6.828911812	6.658346557	6.011324192	6.803293923	0.035663418
NEK8	5.193475644	5.193475644	5.193475644	6.375151524	6.231495476	7.093638396	5.193475644	6.566761799	0.035703493
ARPC5L	5.937471983	6.340620171	5.193475644	6.9226234	6.828911812	6.10435706	5.823855933	6.618630757	0.035876545
TRIM38	6.790137088	6.340620171	5.755880423	7.167912064	7.017227991	6.163617209	6.295545894	6.782919088	0.035966432
MFSD6	5.937471983	6.971474765	6.474542398	5.193475644	6.231495476	5.193475644	6.461163049	5.539482255	0.035973275
PURB	7.080702557	7.416055382	7.391922012	5.193475644	6.344421024	5.193475644	7.29622665	5.577124104	0.036136401
ZBTB16	7.322443252	7.744131107	9.216315484	6.302942904	7.250136145	8.350801506	8.094296614	7.301293518	0.036576114
ITGAL	5.990274404	6.340620171	7.318892736	5.193475644	5.193475644	6.740331914	6.549929104	5.709094401	0.036670625
SIGMAR1	7.52943113	7.408922237	7.7213684	6.302942904	5.193475644	5.193475644	7.553240589	5.563298064	0.036725513
TAC4	5.193475644	5.193475644	5.193475644	6.302942904	6.231495476	7.057644574	5.193475644	6.530694318	0.036910568
AMT	5.193475644	5.193475644	5.193475644	6.302942739	6.231495476	7.057644502	5.193475644	6.530694239	0.036910572
CD84	5.965301214	6.361602266	5.795623904	5.193475644	5.193475644	5.193475644	6.040842461	5.193475644	0.037003789
GSPT1	7.322443252	8.015951925	7.310206594	6.302942904	6.231495476	5.193475644	7.549533924	5.909304675	0.037057495
MLX	6.425901529	7.744131107	7.142215542	5.193475644	5.193475644	5.193475644	7.104082726	5.193475644	0.037543858
DBP	6.425901529	5.193475644	6.159505402	6.9226234	6.231495476	7.057644574	5.926294192	6.737254484	0.037764091
AP2M1	7.710413888	7.408922237	7.310206594	5.193475644	5.193475644	6.10435706	7.47651424	5.497102783	0.037852204
PRPF31	5.937471983	6.971474765	6.474542398	5.193475644	6.231495653	6.10435706	6.461163049	5.843109452	0.037939364
WDR81	5.937471983	6.971475787	6.474542398	5.193475644	6.231495476	6.10435706	6.461163389	5.843109393	0.037939431
SEC24C	5.937471969	6.971476936	6.474542069	5.193475644	6.231495702	6.104356927	6.461163658	5.843109424	0.037939528
EFCAB14	5.937471983	6.340620171	6.732964383	5.193475644	5.193475644	5.193475644	6.337018846	5.193475644	0.038042128
PFKL	5.937471983	6.340620171	6.732964383	5.193475644	5.193475644	5.193475644	6.337018846	5.193475644	0.038042128
RAB5C	5.937471983	6.340620171	6.732964383	5.193475644	5.193475644	5.193475644	6.337018846	5.193475644	0.038042128
SLC25A29	5.937471983	6.340620171	6.732964383	5.193475644	5.193475644	5.193475644	6.337018846	5.193475644	0.038042128
ZC3H4	5.937471983	6.340620171	6.732964383	5.193475644	5.193475644	5.193475644	6.337018846	5.193475644	0.038042128
CANX	5.937471729	6.340620171	6.732964383	5.193475644	5.193475644	5.193475644	6.337018761	5.193475644	0.038042156
PPRC1	6.790137088	6.971474765	6.952053166	5.193475644	5.193475644	6.10435706	6.904555007	5.497102783	0.038573893
PRDX2	7.322443252	6.340620171	6.474542398	5.193475644	5.193475644	5.193475644	6.712535274	5.193475644	0.038593967
RPP14	5.193475644	5.193475644	6.224937831	6.311178532	6.231495476	6.76115876	5.537296373	6.434610923	0.038765235
ZNF264	6.472419761	6.340620171	5.825985195	7.882670108	7.051295819	7.291130399	6.213008376	7.408365442	0.038885509
MAN1B1	5.193475644	6.971474765	6.474542398	7.68677412	8.430313939	7.84450738	6.213164269	7.98719848	0.038909745
SBF1	6.425901529	7.408922237	7.142215542	5.193475644	6.828911812	6.10435706	6.992346436	6.042248172	0.039018805
ZNF324	5.937471983	5.193475644	6.159505402	6.9226234	6.231495476	6.658346557	5.763484343	6.604155144	0.039228347
SYNPO2L	6.808712565	6.971474765	6.035766751	5.193475644	5.193475644	5.193475644	6.605318027	5.193475644	0.039342587
PGAM5	5.937471983	6.340620171	6.754209954	5.193475644	5.193475644	5.193475644	6.344100703	5.193475644	0.03951722
MYO5B	7.080702557	6.340620171	5.755880423	6.302942904	5.193475644	5.193475644	6.39240105	5.563298064	0.039888711
ARID1A	9.116716401	9.154822291	9.619695644	6.302942904	7.575763645	8.033708627	9.297078112	7.304138392	0.039889994
SNX22	8.773560935	8.992836841	9.417866768	10.08052024	9.61497833	10.36453715	9.061421515	10.02001191	0.040030657
PCDH11Y	6.645152109	5.193475644	5.193475644	7.619598573	7.284590277	7.004901804	5.677367799	7.303030218	0.040043356
GHRL	5.193475644	5.193475644	5.193475644	6.922621385	6.231495476	6.10435706	5.193475644	6.419491307	0.040410245
ERAP1	5.193475644	5.193475644	5.193475644	6.9226234	6.231495476	6.10435706	5.193475644	6.419491979	0.040410403
NDUFA6	5.193475644	5.193475644	5.193475644	6.922623904	6.231495476	6.10435706	5.193475644	6.419492147	0.040410442
BAG6	9.346736476	7.40892186	8.302444479	6.922624408	6.231496298	6.658347017	8.352700938	6.604155908	0.040636045
GOSR2	5.937471983	6.340620171	5.764373789	5.193475644	5.193475644	5.193475644	6.014155314	5.193475644	0.040648217
NUDT17	5.193475644	5.193475644	5.193475644	8.183696709	8.259366931	6.658346557	5.193475644	7.700470066	0.040654033
TEX101	5.956436231	6.340620171	5.755880423	5.193475644	5.193475644	5.193475644	6.017645608	5.193475644	0.040700057
ACTB	8.355165753	9.458107057	8.978947505	7.48830238	7.841259061	8.033708627	8.930740105	7.787756689	0.040731226
PLEKHG6	6.425901529	6.340620171	5.777027698	6.9226234	6.828911827	6.658346563	6.181183132	6.80329393	0.040779177
URB1	6.425901529	6.340620171	5.81559751	6.670664699	6.856038641	6.312783353	6.194039736	6.613162231	0.040785694
BAIAP2L1	6.425901529	5.193475644	6.474542398	7.354711538	6.828911812	7.370045892	6.031306524	7.184556414	0.041094273
KPNA2	6.425901529	6.971474765	6.952053166	5.193475644	5.193475644	6.10435706	6.783143153	5.497102783	0.041330529
PTGFR	5.193475644	5.193475644	5.193475644	7.354711035	6.231495759	6.658344943	5.193475644	6.748183912	0.041584892
GSDME	5.193475644	5.193475644	5.193475644	7.354711538	6.231495476	6.658346557	5.193475644	6.748184524	0.041584899
RAB26	5.193475644	5.193475644	6.474542398	6.9226234	6.231495476	7.370047178	5.620497896	6.841388685	0.041700553
XIAP	7.330659631	7.358447028	7.155402068	7.743511829	8.119209835	8.062010298	7.281502909	7.974910654	0.041833204
ACY1	5.937471983	6.340620171	5.755880423	5.193475644	5.193475644	5.193475644	6.011324192	5.193475644	0.041854953
ARHGEF16	5.937471983	6.340620171	5.755880423	5.193475644	5.193475644	5.193475644	6.011324192	5.193475644	0.041854953
ARHGEF37	5.937471983	6.340620171	5.755880423	5.193475644	5.193475644	5.193475644	6.011324192	5.193475644	0.041854953
ATP6V0C	5.937471983	6.340620171	5.755880423	5.193475644	5.193475644	5.193475644	6.011324192	5.193475644	0.041854953
BIK	5.937471983	6.340620171	5.755880423	5.193475644	5.193475644	5.193475644	6.011324192	5.193475644	0.041854953
CAD	5.937471983	6.340620171	5.755880423	5.193475644	5.193475644	5.193475644	6.011324192	5.193475644	0.041854953
CAT	5.937471983	6.340620171	5.755880423	5.193475644	5.193475644	5.193475644	6.011324192	5.193475644	0.041854953
CHID1	5.937471983	6.340620171	5.755880423	5.193475644	5.193475644	5.193475644	6.011324192	5.193475644	0.041854953
FAM118A	5.937471983	6.340620171	5.755880423	5.193475644	5.193475644	5.193475644	6.011324192	5.193475644	0.041854953
FBXO10	5.937471983	6.340620171	5.755880423	5.193475644	5.193475644	5.193475644	6.011324192	5.193475644	0.041854953
HYAL4	5.937471983	6.340620171	5.755880423	5.193475644	5.193475644	5.193475644	6.011324192	5.193475644	0.041854953
KAZN	5.937471983	6.340620171	5.755880423	5.193475644	5.193475644	5.193475644	6.011324192	5.193475644	0.041854953
LYPLAL1	5.937471983	6.340620171	5.755880423	5.193475644	5.193475644	5.193475644	6.011324192	5.193475644	0.041854953
MAGEA3	5.937471983	6.340620171	5.755880423	5.193475644	5.193475644	5.193475644	6.011324192	5.193475644	0.041854953
MAP4K4	5.937471983	6.340620171	5.755880423	5.193475644	5.193475644	5.193475644	6.011324192	5.193475644	0.041854953
MAPK14	5.937471983	6.340620171	5.755880423	5.193475644	5.193475644	5.193475644	6.011324192	5.193475644	0.041854953
PPP2R5B	5.937471983	6.340620171	5.755880423	5.193475644	5.193475644	5.193475644	6.011324192	5.193475644	0.041854953
PPWD1	5.937471983	6.340620171	5.755880423	5.193475644	5.193475644	5.193475644	6.011324192	5.193475644	0.041854953
PRKD3	5.937471983	6.340620171	5.755880423	5.193475644	5.193475644	5.193475644	6.011324192	5.193475644	0.041854953
PROSER1	5.937471983	6.340620171	5.755880423	5.193475644	5.193475644	5.193475644	6.011324192	5.193475644	0.041854953
RAB11FIP5	5.937471983	6.340620171	5.755880423	5.193475644	5.193475644	5.193475644	6.011324192	5.193475644	0.041854953
RARG	5.937471983	6.340620171	5.755880423	5.193475644	5.193475644	5.193475644	6.011324192	5.193475644	0.041854953
RRAGC	5.937471983	6.340620171	5.755880423	5.193475644	5.193475644	5.193475644	6.011324192	5.193475644	0.041854953
SEZ6L2	5.937471983	6.340620171	5.755880423	5.193475644	5.193475644	5.193475644	6.011324192	5.193475644	0.041854953
SMCHD1	5.937471983	6.340620171	5.755880423	5.193475644	5.193475644	5.193475644	6.011324192	5.193475644	0.041854953
SNX14	5.937471983	6.340620171	5.755880423	5.193475644	5.193475644	5.193475644	6.011324192	5.193475644	0.041854953
SSBP3	5.937471983	6.340620171	5.755880423	5.193475644	5.193475644	5.193475644	6.011324192	5.193475644	0.041854953
ST13	5.937471983	6.340620171	5.755880423	5.193475644	5.193475644	5.193475644	6.011324192	5.193475644	0.041854953
TATDN1	5.937471983	6.340620171	5.755880423	5.193475644	5.193475644	5.193475644	6.011324192	5.193475644	0.041854953
TBCK	5.937471983	6.340620171	5.755880423	5.193475644	5.193475644	5.193475644	6.011324192	5.193475644	0.041854953
TMEM191B	5.937471983	6.340620171	5.755880423	5.193475644	5.193475644	5.193475644	6.011324192	5.193475644	0.041854953
TTC23	5.937471983	6.340620171	5.755880423	5.193475644	5.193475644	5.193475644	6.011324192	5.193475644	0.041854953
WNK2	5.937471983	6.340620171	5.755880423	5.193475644	5.193475644	5.193475644	6.011324192	5.193475644	0.041854953
ZNF595	5.937471983	6.340620171	5.755880423	5.193475644	5.193475644	5.193475644	6.011324192	5.193475644	0.041854953
ZNF610	5.937471983	6.340620171	5.755880423	5.193475644	5.193475644	5.193475644	6.011324192	5.193475644	0.041854953
TRO	5.937471983	6.340620353	5.755880423	5.193475644	5.193475644	5.193475644	6.011324253	5.193475644	0.041854974
C1orf198	5.937471983	6.340620546	5.755880423	5.193475644	5.193475644	5.193475644	6.011324317	5.193475644	0.041854995
STARD13	5.937471983	6.340620221	5.755880001	5.193475644	5.193475644	5.193475644	6.011324068	5.193475644	0.04185502
TP53I11	7.080702557	6.340620171	6.159505402	5.193475644	5.193475644	5.193475644	6.52694271	5.193475644	0.041867164
TRIO	7.080702557	6.340620171	6.159505402	5.193475644	5.193475644	5.193475644	6.52694271	5.193475644	0.041867164
ZEB1	7.322777073	6.340620171	7.142215542	6.302942904	5.193475644	5.193475644	6.935204262	5.563298064	0.04209865
MUTYH	8.015854714	7.744131107	8.040966072	6.302942904	6.828911812	7.057644574	7.933650631	6.729833097	0.042154182
TLL2	5.937471983	5.193475644	6.474542398	6.9226234	7.250136145	7.84450738	5.868496675	7.339088975	0.042535235
FBXO44	5.982835618	6.551299951	5.904483828	5.193475644	5.193475644	5.193475644	6.146206466	5.193475644	0.042841436
ZBTB45	9.775037593	10.16473129	10.58574561	10.79231849	10.65911525	11.24080343	10.1751715	10.89741239	0.042914171
BRICD5	5.193475644	5.193475644	5.193475644	6.302942904	7.121137366	6.210308393	5.193475644	6.544796221	0.042935224
RAPGEF1	6.425901736	6.340620068	5.755880647	5.193475644	5.193475644	5.193475644	6.17413415	5.193475644	0.043144442
TMEM9	6.425901389	6.340619971	5.755880423	5.193475644	5.193475644	5.193475644	6.174133928	5.193475644	0.043144457
BICD2	6.425901529	6.340620171	5.755880423	5.193475644	5.193475644	5.193475644	6.174134041	5.193475644	0.043144468
E2F7	6.425901529	6.340620171	5.755880423	5.193475644	5.193475644	5.193475644	6.174134041	5.193475644	0.043144468
GAS2L1	6.425901529	6.340620171	5.755880423	5.193475644	5.193475644	5.193475644	6.174134041	5.193475644	0.043144468
INTS4	6.425901529	6.340620171	5.755880423	5.193475644	5.193475644	5.193475644	6.174134041	5.193475644	0.043144468
L3MBTL2	6.425901529	6.340620171	5.755880423	5.193475644	5.193475644	5.193475644	6.174134041	5.193475644	0.043144468
LZTS2	6.425901529	6.340620171	5.755880423	5.193475644	5.193475644	5.193475644	6.174134041	5.193475644	0.043144468
MRPS12	6.425901529	6.340620171	5.755880423	5.193475644	5.193475644	5.193475644	6.174134041	5.193475644	0.043144468
NBPF1	6.425901529	6.340620171	5.755880423	5.193475644	5.193475644	5.193475644	6.174134041	5.193475644	0.043144468
NCSTN	6.425901529	6.340620171	5.755880423	5.193475644	5.193475644	5.193475644	6.174134041	5.193475644	0.043144468
PSMD7	6.425901529	6.340620171	5.755880423	5.193475644	5.193475644	5.193475644	6.174134041	5.193475644	0.043144468
REPS1	6.425901529	6.340620171	5.755880423	5.193475644	5.193475644	5.193475644	6.174134041	5.193475644	0.043144468
Sep-07	6.425901529	6.340620171	5.755880423	5.193475644	5.193475644	5.193475644	6.174134041	5.193475644	0.043144468
SIAH2	6.425901529	6.340620171	5.755880423	5.193475644	5.193475644	5.193475644	6.174134041	5.193475644	0.043144468
UNK	6.425901529	6.340620171	5.755880423	5.193475644	5.193475644	5.193475644	6.174134041	5.193475644	0.043144468
XKR5	6.425901529	6.340620171	5.755880423	5.193475644	5.193475644	5.193475644	6.174134041	5.193475644	0.043144468
PPP2R5E	6.425901649	6.340620171	5.755880423	5.193475644	5.193475644	5.193475644	6.174134081	5.193475644	0.043144474
DEPTOR	6.425902491	6.340620171	5.755880423	5.193475644	5.193475644	5.193475644	6.174134362	5.193475644	0.043144515
TAP2	6.42590244	6.340620171	5.755879958	5.193475644	5.193475644	5.193475644	6.17413419	5.193475644	0.043144585
ZFP14	6.632263656	6.889821398	7.130079871	6.974635526	7.225056831	7.747106082	6.884054975	7.31559948	0.043232359
ZNF224	5.193475644	6.971474765	6.159505402	6.302942904	7.841259061	6.658346557	6.108151937	6.934182841	0.043261248
MST1	6.425901529	6.340620171	6.159505402	6.9226234	7.250136145	6.658346557	6.308675701	6.943702034	0.043673512
C14orf119	5.937471983	5.193475644	6.474542398	6.9226234	6.907729702	7.370045892	5.868496675	7.066799665	0.043763461
DHFR	5.937471983	5.193475644	5.193475644	7.354711538	6.231495476	7.370045892	5.441474424	6.985417635	0.043926906
ARL6IP4	6.425901529	6.340620171	5.755880423	6.9226234	6.828911812	6.658346557	6.174134041	6.803293923	0.044092833
CDK10	7.017725555	6.340620171	6.952053166	7.821391851	8.06540678	8.108361568	6.770132964	7.998386733	0.044559898
PMS2	9.116718326	9.363922678	8.774195141	8.706831987	8.583134117	7.84450738	9.084945382	8.378157828	0.044636508
PGAM1	7.080702557	6.971474765	6.952053166	5.193475644	5.193475644	6.10435706	7.001410163	5.497102783	0.044859816
STIP1	7.080702557	8.015951925	7.7213684	6.302942904	7.250136145	7.370045892	7.606007627	6.97437498	0.045900941
CCDC18	7.871202567	7.744131107	8.302444768	8.8461452	8.583134117	8.724835423	7.972592814	8.718038247	0.046298069
ZNF714	6.949367825	7.228837394	6.732964383	5.193475644	6.473137037	5.193475644	6.970389867	5.620029442	0.047076009
PLA2R1	5.193475644	5.193475644	5.781994315	6.302942904	7.330249714	6.957099839	5.389648534	6.863430819	0.047193932
CLEC2D	5.937471983	5.193475644	6.357180008	7.354711538	6.828911812	7.057644574	5.829375878	7.080422641	0.047365263
CCND1	10.19533198	10.59611375	10.76997995	9.48054548	8.963341285	9.602490318	10.52047523	9.348792361	0.047537968
IDH2	6.892420372	7.443249556	6.159505402	5.193475644	5.193475644	5.193475644	6.83172511	5.193475644	0.047846312
UNC13A	6.790137088	6.340620171	6.159505402	6.302942904	5.193475644	5.193475644	6.430087554	5.563298064	0.047907895
SMARCC2	6.425901529	6.340620171	6.474541696	7.354711538	6.828911812	7.626693983	6.413687799	7.270105778	0.048144677
ZNF512B	6.790137088	6.340620171	7.142215542	6.302942904	5.193475644	6.10435706	6.7576576	5.866925203	0.048753798
USP9X	6.790137088	6.971474765	6.17487604	5.193475644	6.231495476	5.193475644	6.645495965	5.539482255	0.049273114
NUTM2B	5.193475644	5.193475644	5.440541385	7.68677412	6.236430953	7.562223792	5.275830891	7.161809622	0.049287579
E2F4	5.937471983	6.971474765	6.732964383	5.193475644	5.193475644	5.193475644	6.54730371	5.193475644	0.049395937
HIST1H3B	5.937471983	6.971474765	6.732964383	5.193475644	5.193475644	5.193475644	6.54730371	5.193475644	0.049395937
LRP5	5.937471983	6.971474765	6.732964383	5.193475644	5.193475644	5.193475644	6.54730371	5.193475644	0.049395937
SSC4D	6.790137088	6.340620171	6.159505402	7.956525514	6.828911812	7.057644574	6.430087554	7.2810273	0.049717515
ITGA7	8.015854714	8.244587753	8.220442032	7.68677412	7.575763645	7.84450738	8.160294833	7.702348381	0.049886307
C11orf95	6.884868097	7.408922237	7.310206594	6.302942904	6.828911812	7.057644574	7.201332309	6.729833097	0.049904197
CRX	5.193475644	5.193475644	5.296691474	6.966160375	6.29844447	6.10435706	5.227880921	6.456320635	0.04994425
KRIT1	5.937471983	5.193475644	6.159505402	6.9226234	6.828911812	6.92080479	5.763484343	6.890780001	0.050056281
RAB10	6.790137088	6.971474765	6.159505402	5.193475644	6.231495476	5.193475644	6.640372419	5.539482255	0.050169446
RAB13	6.790137088	7.408922237	7.142215542	6.302942904	6.231495476	6.10435706	7.113758289	6.212931813	0.050593577
B3GAT2	9.555907168	9.629821906	9.799900407	8.552613963	7.841259061	8.92929174	9.661876494	8.441054921	0.050886377
POFUT1	5.937471983	6.971474765	7.835949747	5.193475644	5.193475644	6.541827132	6.914965498	5.64292614	0.050960412
RACK1	6.790137088	7.408922237	7.7213684	6.302942904	6.231495476	6.658346557	7.306809242	6.397594979	0.050996155
BBS5	5.193475644	5.193475644	7.09398605	8.390926845	7.17750193	8.536052846	5.826979113	8.034827207	0.051052897
ASF1B	5.937471983	6.971474765	6.474542398	5.193475644	5.193475644	5.193475644	6.461163049	5.193475644	0.051243398
COPG1	5.937471983	6.971474765	6.474542398	5.193475644	5.193475644	5.193475644	6.461163049	5.193475644	0.051243398
NAA60	5.937471983	6.971474765	6.474542398	5.193475644	5.193475644	5.193475644	6.461163049	5.193475644	0.051243398
WSB2	7.275901735	7.744131107	6.971027777	5.193475644	6.231495476	6.10435706	7.330353539	5.843109393	0.051490312
C11orf54	5.193475644	6.340620171	5.193475644	6.949707039	7.250136145	6.10435706	5.575857153	6.768066748	0.051656307
ATAD2	6.425901529	6.971474765	5.755880423	5.193475644	6.231495476	5.193475644	6.384418906	5.539482255	0.051917212
STAC2	5.193475644	5.193475644	5.193475644	7.08007894	6.231495476	6.109864415	5.193475644	6.473812944	0.052383823
ABCA9	6.790137088	5.193475644	6.159505402	7.68677412	7.398656233	7.84450738	6.047706045	7.643312577	0.05240347
EIF2S3	6.986174497	6.340620171	5.972468073	5.193475644	5.193475644	5.193475644	6.433087581	5.193475644	0.052648638
MAFK	5.937471983	6.971474765	6.952053166	5.193475644	5.193475644	5.193475644	6.620333305	5.193475644	0.052780911
ENTPD1	7.107710389	6.873572088	6.952053166	7.49705719	7.841259061	7.719826702	6.977778548	7.686047651	0.052826423
MASTL	5.193475644	6.340620171	5.193475644	7.354711538	7.250136145	6.658346557	5.575857153	7.087731413	0.052856526
CARD9	5.937471983	5.193475644	6.952053166	7.354711538	7.575763645	8.033708627	6.027666931	7.654727937	0.052894049
CASZ1	7.322443029	7.40892035	6.474541266	6.302942904	5.193475644	5.193475644	7.068634882	5.563298064	0.053522097
KDM4B	6.425901529	7.199333841	7.310206594	5.193475644	6.231495476	5.193475644	6.978480655	5.539482255	0.053627345
APC2	9.051075965	8.441897719	8.884810686	8.552613963	7.575763645	8.486542743	8.79259479	8.20497345	0.053891712
MZT2B	5.937471983	6.340620171	6.952053166	5.193475644	5.193475644	5.193475644	6.41004844	5.193475644	0.054051574
PABPC1	5.937471983	6.340620171	6.952053166	5.193475644	5.193475644	5.193475644	6.41004844	5.193475644	0.054051574
SLC9A3R1	5.937471983	6.340620171	6.952053166	5.193475644	5.193475644	5.193475644	6.41004844	5.193475644	0.054051574
C7orf50	5.937471983	6.971474765	6.372271425	5.193475644	5.193475644	5.193475644	6.427072724	5.193475644	0.054279404
ZBTB5	5.937471983	6.340620171	7.142215542	5.193475644	5.193475644	6.658346557	6.473435898	5.681765949	0.054583507
MKNK2	7.52943113	8.015951925	7.835949747	6.302942904	5.193475644	6.10435706	7.793777601	5.866925203	0.054868504
IRF2BPL	8.143554023	6.35664159	7.46066066	5.193475644	5.193475644	5.193475644	7.320285424	5.193475644	0.055017022
SH3BP5	6.425901529	5.193475644	6.952053166	7.68677412	7.250136145	7.84450738	6.19047678	7.593805881	0.055029492
NAPEPLD	6.790137088	6.971474765	6.474542398	7.354711538	7.575763645	7.626693983	6.74538475	7.519056388	0.055129881
CCDC80	7.080702557	6.340620171	7.151347701	8.421762669	7.601235627	7.685199083	6.85755681	7.90273246	0.055365862
RABL6	10.16462335	9.986566358	10.60203109	10.85964483	10.3107154	11.43791517	10.2510736	10.86942513	0.055861865
HJURP	9.015751386	7.408922237	6.245608382	6.302942904	5.193475644	5.193475644	7.556760668	5.563298064	0.055874624
LSMEM2	5.937471983	7.408922237	6.732964383	6.9226234	7.841259061	7.84450738	6.693119534	7.536129947	0.056101541
NOMO1	5.937471983	6.971475276	6.3260105	5.193475644	5.193475644	5.193475644	6.411652586	5.193475644	0.056163342
XRCC2	7.070187364	7.744131107	7.486763456	7.458792576	8.043155416	8.158205905	7.433693976	7.886717965	0.056244061
KDM6B	8.578536936	8.244587753	9.475118761	7.354711538	5.193475644	7.370045892	8.76608115	6.639411025	0.056392107
ABRAXAS1	5.193475644	6.971474765	6.159505402	6.925150099	7.659726701	7.844506773	6.108151937	7.476461191	0.056646248
PRKDC	6.790137088	6.340620171	6.732964383	5.193475644	5.193475644	6.10435706	6.621240548	5.497102783	0.056690545
LRG1	5.937471983	5.193475644	5.193475644	6.357744652	6.289507266	6.10435706	5.441474424	6.250536326	0.056851064
PPIA	8.106858879	6.340620171	7.835949747	6.922623904	5.193475644	7.370045892	7.427809599	6.495381813	0.0573711
RHBDD2	6.425901529	8.441897719	7.835949747	5.193475644	5.193475644	5.193475644	7.567916332	5.193475644	0.057825647
HUS1	5.937471983	7.113828733	5.756113275	5.193475644	6.828911812	5.193475644	6.269137997	5.738621034	0.057875798
ZCCHC3	7.080702557	8.244587753	8.523724941	6.302942904	6.231495476	7.057644574	7.94967175	6.530694318	0.057973168
HECA	6.790137088	6.421947526	6.014979444	6.302942904	5.193475644	5.193475644	6.409021353	5.563298064	0.058634335
DYNLL2	6.425901529	6.340620171	7.596895559	5.193475644	5.193475644	5.193475644	6.787805753	5.193475644	0.058964531
TESK2	6.790137088	5.193475644	5.193475644	7.354711538	6.231495476	6.658346557	5.725696126	6.748184524	0.05899905
CPN2	5.193475644	5.193475644	6.333092505	6.9226234	6.231495476	7.057644574	5.573347931	6.737254484	0.059284544
SLC46A1	9.602476947	9.546517741	10.17124258	10.56914364	10.69310176	10.5984166	9.773412422	10.62022067	0.05940712
WDR88	5.937471983	6.340620171	6.474542398	6.302942904	6.828911812	6.658346557	6.250878184	6.596733758	0.059607443
NCOR2	7.529431323	7.408921294	6.732965282	6.302942796	6.828914209	6.104357397	7.223772633	6.412071467	0.05976289
KIF18B	5.937471983	6.817608909	7.21218206	5.193475644	5.193475644	5.193475644	6.655754317	5.193475644	0.060433149
FAM228B	5.193475644	5.193475644	5.193475644	7.68677412	6.231495476	6.658346557	5.193475644	6.858872051	0.061152053
INTS6	8.690719488	8.353531137	7.985373822	7.956525514	7.434287133	7.650071522	8.343208149	7.680294723	0.061396262
FGF22	5.193475644	5.193475644	5.193475644	6.302942634	7.25013466	6.104357075	5.193475644	6.552478123	0.061488956
